# Do we need to “man up” feeding and eating disorders treatments? Protocol for a systematic review and individual patient data meta-analysis of gender effects on intervention outcomes

**DOI:** 10.1186/s13643-025-03041-5

**Published:** 2025-12-24

**Authors:** Georg Halbeisen, Nina Timmesfeld, Georgios Paslakis

**Affiliations:** 1 University Clinic for Psychosomatic Medicine and Psychotherapy, Medical Faculty, Campus East-Westphalia, Ruhr-University Bochum, Virchowstr. 65, Luebbecke, 32312 Germany; 2 Department of Medical Informatics, Biometrics and Epidemiology, Ruhr-University Bochum, Universitaetsstraße 105, 44789 Bochum, Germany

**Keywords:** Anorexia nervosa, Bulimia nervosa, Binge-eating disorder, OSFED, ARFID, Psychotherapy

## Abstract

**Background:**

Feeding and eating disorders lead to serious health impairments. Boys and men are increasingly affected and may account for a fourth of clinical cases. Current evidence suggests that symptoms and health care needs differ between men and women (e.g., related to muscularity concerns), and that men delay seeking treatment due to the traditional understanding of eating disorders as “women’s disease”. Treatment guidelines recommend psychotherapy as first-line intervention, but potential gender differences in treatment responses have not been considered. This is due to the lack of systematic evaluations of gender differences related to treatment outcomes for feeding and eating disorders.

**Methods:**

This systematic review with Individual Patient Data Meta-Analysis (IPDMA) will compare the effects of psychotherapeutic interventions for feeding and eating disorders between gender groups. The focus of the analysis is on eating disorders-related psychopathology. For this purpose, randomized-controlled clinical studies will be identified in scientific databases and examined for their methodological quality. Primary investigators will be contacted to deliver anonymized data of their studies. Study results will then be summarized and compared in a two-staged IPDMA. Gender groups will be compared regarding different types of interventions and further relevant intervention characteristics, as identified by men with lived experience.

**Discussion:**

The results will help to formulate treatment recommendations and identify the treatment contexts that are associated with unfavorable outcomes related to patient gender. This may fuel efforts of adapting established interventions to best meet the health care needs of men and women in the context of eating disorders.

**Systematic review registration:**

PROSPERO CRD42022372712.

**Supplementary Information:**

The online version contains supplementary material available at 10.1186/s13643-025-03041-5.

## Introduction

### Rationale

Feeding and eating disorders (FEDs) are characterized by body image concerns, altered eating patterns, and weight-control behaviors [1]. The most common FEDs based on lifetime estimates are binge-eating disorder (BED, up to 2.8%), bulimia nervosa (BN, 1.9%), anorexia nervosa (AN, 1.4%), and other specified feeding or eating disorder (OSFED, 4.3%), with limited data available for more recently specified diagnoses, e.g., avoidant/restrictive food intake disorder (ARFID) [2, 3]. Point prevalence for FEDs among the general population increased between 2000 and 2018, from 3.5 to 7.8%, with subclinical pathology reaching 17% [4]; associated symptoms (e.g., body dissatisfaction) affect as many as 48.3% women and 33.2% men [5]. FEDs pose one of the highest mortality risks among mental disorders. Compared to the general population, all-cause mortality risk is 5.9 times higher for patients with AN, and 1.9 times higher for patients with BN or other FEDs [6]. Comorbidities are frequent, as exemplified in two recent systematic reviews and meta-analyses by our research group on FEDs and behavioral addictions [7] or substance abuse [8]. The associated treatment and economic costs are substantial [9], with annual rates in Germany due to treatment and lost productivity for AN and BN alone amounting to 319 Mio. € [10].

Although more common in girls and women, FEDs are increasingly recognized as a health risk in boys and men. In fact, evidence points to an unparalleled increase, as boys and men could make up for 25% of clinical FED cases [11]. In a recent North American community sample of boys and men, FED prevalence was 21.3% [12]. Similarly, and although FEDs pose a higher burden for women compared to men (60.46 vs. 26.66 disability-adjusted life years, DALYs), men’s burden has been increasing comparably faster over the last 30 years (0.70% vs. 0.63% annual increase) [13]. However, men remain underrepresented in FED research [14], including clinical trials [15]. It is therefore unknown, if the currently available psychological interventions, recommended as first-line interventions in several international guidelines due to their demonstrable efficacy [16], are equally effective in reducing FED pathology across genders, creating uncertainty among clinicians about FED treatment implementation.

### Gender differences in FED presentation and treatment

FEDs have traditionally been perceived as only affecting girls and women, as illustrated by former diagnostic criteria being inapplicable to boys and men (e.g., amenorrhea in the case of AN) [17], and by diagnostic questionnaires geared toward FED presentation in girls and women [18]. For example, men with FEDs can be less concerned with thinness [19], often seek to increase muscularity [20], and also show different patterns in emotion regulation associated with FED pathology [21]. Recent network analyses support the assumed centrality of muscularity concerns in men’s symptom networks [22]. As a result, considerable effort has recently been devoted to further the understanding of FED presentation [23] and etiology [24] in boys and men, and to adjusting diagnostic criteria [25] and tools [26, 27].

However, specific treatment needs of boys and men with FEDs have not been adequately addressed [28], as illustrated by our group’s systematic review of qualitative studies [29]. Men are subjected to social stigma related to the perceived “femininity” of FEDs [30], often delay help-seeking, and present later in the course of their illness, when psychopathology is more severe and less tractable to intervention [31]. Recent quantitative evidence also showed that perceptions of FEDs as “women’s diseases” are associated with reduced help‑seeking intentions in men [32]. Moreover, men with FEDs face unique challenges during treatment, and often report struggling to feel understood by therapists [33], feeling unwanted and that their concerns are not taken into account [34]. These reports and data suggest that men’s under-representation in FED research may have inadvertently facilitated the development of women-centered FED treatments [35], leading to suboptimal treatment experiences for men.

Due to the lack of gender-based subgroup comparisons in existing reviews and meta-analyses, uncertainty remains, whether and to what extent reports of negative treatment experiences are associated with gender-based differences in FED treatment outcomes. In the past, authors noted that relevant primary research was non-existent [36], but men are included in FED trials and recent years also saw more interest in conducting gender-specific comparisons. For example, our group recently compared men and women with FEDs with regard to clinical phenotypes, therapy response, and drop outs [19, 37–40], finding that men and women differ in weight gains during treatment as well as changes in FED-specific and general psychopathology [41]. Another recent study found men with FEDs to present with more severe symptoms, but to also show a tendency for greater improvement at discharge [42]. Nonetheless, there is a need to systematically summarize, synthesize, and evaluate existing data for gender disparities and/or equivalence, as the evidence on treatment effects in single publications appears ambiguous. For example, whereas another study also found comparable outcomes for BN, and even better outcomes for AN when comparing long-term outcomes between men and women who had undergone treatment [43], an analysis of aggregated RCT data from a single medical center found no gender-related differences in BED treatment outcomes [44]. Although important, the individual analyses may be considered inconclusive, as they neither systematically explore the available evidence in its entirety, nor address specific aspects like treatment type (e.g., outcomes of CBT vs. other first-line treatments) and its relation to gender-specific aspects.

Recognizing the need for investigating FED treatment outcomes in boys and men, Hendricks et al. recently published a systematic review on the effects of psychological interventions for improving body image and reducing disordered eating in adult men [45]. However, the review did not include clinical samples, and the authors explicitly refrained from quantifying gender differences in treatment outcomes. Of course, such comparisons are absent from many psychotherapy-centered RCTs [46], which questions the feasibility of only reviewing aggregated data. To overcome this limitation, we pursue to combine the large volume of individual trial data in a systematic review with Individual Patient Data Meta-Analysis (IPDMA).

### Objectives

Because girls and women constitute a majority within RCT samples [15], it is currently unknown, if the currently available psychological interventions are equally effective in reducing FED psychopathology across genders, creating uncertainty for clinicians about FED treatment implementation. Thus, the objective of this IPDMA is to examine, whether gender, in patients with FEDs, moderates the effect of psychological interventions, compared to active or passive controls, on improving FED psychopathology. Given the paucity of conclusive evidence from individual studies, we refrained from formulating a directional hypothesis concerning the effect of gender.

## Methods

### Eligibility criteria

Studies that meet the following criteria will be considered eligible for inclusion: (a) psychological treatment studies that examined manualized or non-manualized, therapist-led, internet-based, or self-help psychological interventions for FEDs as recommended in international guidelines [16], that (b) included men and women patients with a clinical FED diagnosis according to DSM-IV, DSM-V, or ICD-10 criteria (e.g., anorexia nervosa [AN], bulimia nervosa [BN], other specified feeding and eating disorder [OSFED], avoidant/restrictive food intake disorder [ARFID], binge-eating disorder [BED]), (c) used an RCT design with active (alternative interventions, treatment-as-usual) or passive (waitlist) control groups, (d) assessed FED-associated psychopathology, (e) included a baseline, an end-of-treatment, and/or at least one follow-up assessment, and (f) included at least one validated primary or secondary outcome measure (see below).

We will exclude (a) double reports of the same trial, (b) studies with non-clinical or sub-clinical cohorts (e.g., student samples, community samples), and (c) solely pharmacological trials.

### Information sources

We will conduct an electronic search in accordance with the Peer Review of Electronic Search Strategies (PRESS) guideline for systematic reviews [47]. The following electronic databases will be included in the search: ANNUAL REVIEWS, Cochrane Database of Systematic Reviews, DARE, JSTOR, LILACS, MEDLINE (via PubMed), NIHR Centre for Reviews and Dissemination, PsycArticles, PsychINFO, Psyndex plus, Catalogue of the German National Library of Medicine (via LIVIVO), PubPSYCH, Scopus, Web of Science. In addition, national and international trial registers will be searched: CENTRAL, Clinical Trials.gov, Deutsches Register Klinischer Studien, EU Clinical Trials Register, U.K. Clinical Trials Gateway, WHO International Clinical Trials Registry Platform. There will be no restrictions placed on year of publication, publication type, or language of the full record (however, for the assessment of eligibility, an English abstract must be available). The reference lists of any included articles will be individually searched for relevant studies not found through searching the online databases. We will also ask experts in the field to identify other published or ongoing trials. Potentially eligible trials that are not yet completed will not be included in this IPDMA, but will be noted for inclusion in future updates.

### Search strategy

Natural language terms and, where applicable, Medical Subject Headings (MeSH) concerning relevant populations (e.g., anorexia nervosa), interventions (e.g., Cognitive Behavioral Therapy), and outcomes (e.g., FED psychopathology) were used to construct broad search strings (see Additional file 1).

### Data management and selection process

All identified records will be transferred into the Covidence review software. After removing duplicates, two investigators with relevant expertise (e.g., psychologists, M.Sc. level or PhD) will independently screen titles, abstracts, and full-texts, if needed. Full-texts of all selected articles will then be reviewed independently by the two reviewers to determine inclusion in the systematic review and IPDMA in accordance with selection criteria. Disagreement between reviewers after full-text review will be resolved through consulting with the principal investigator.

### Data collection process

The principal investigator and the cooperating partners will contact and invite principal investigators (PIs) of eligible RCTs to join the consortium (preliminary labelled “MAN-UP IPDMA”) and to share their data. In case of no response, we will send reminders after 4 and 8 weeks, search for alternative addresses, and contact another investigator of the same study. After PIs express their interest in data sharing, they will be requested to sign a data sharing agreement and to supply anonymized trial data. The various data formats will be checked for completeness, improbable values, consistency with published articles, and missing items. Actual data coding and transfer from original studies into the IPD database will be done by a supervised staff or trainee member of the team. Participant characteristics and screening accuracy results for each study using the cleaned datasets will be compared with those from the original datasets to identify any potential discrepancies.

### Data items

The data that will be requested and/or extracted include: title, reference, publication year, publication type, funding sources and conflicts of interest, type of study, study design and aim, participant information (e.g., diagnosis, gender, age), recruitment process, intervention setting, type of intervention, outcome measures, risk of bias, statistical methods, pre-post measurement intervals, key results, authors´ conclusions, and data for the primary and secondary endpoints. Additional data items may be formulated.

### Outcomes and prioritization

Pre- and post (end-of-treatment)-intervention FED psychopathology assessed by the Eating Disorder Examination-Questionnaire (EDE-Q), the Eating Disorder Inventory-2 (EDI-2), or comparable standardized diagnostic assessments of core FED symptoms, will serve as primary outcome of treatment effects. Secondary outcomes may include pre- and post-intervention FED-related psychopathology (e.g., depressive symptoms, quality of life/health-related quality of life), body mass index, and treatment-related aspects (e.g., dropout rates, remission), if reported.

As part of our protocol preparation, we specifically asked men with FEDs which treatment characteristics they perceive as relevant for treatment outcomes in men. The interviewed men recommended to stratify treatment outcomes by factors related to setting characteristics (i.e., group vs. individual therapy, in- vs. outpatient treatment) for the purposes of the planned IPDMA. Treatment approaches (i.e., CBT vs. psychodynamic therapy) were ranked secondary in importance. Features connected to health professionals per se (e.g., therapists’ gender) were considered least important during inpatient treatment, but nevertheless relevant for seeking initial access to outpatient care. The planned data extraction and moderator analyses accommodate these recommendations, if feasible. Additional input will be sought from stakeholders prior to data extraction.

### Risk of bias in individual studies

Two reviewers with methodological expertise (psychologist, M.Sc. level or PhD; statistician) will independently assess the risk of bias using the Cochrane revised risk-of-bias (RoB 2) tool for RCTs [48].

### Data synthesis

The systematic review with IPD meta-analysis will investigate the efficacy of psychological interventions for improving FED psychopathology in men and women with FEDs. First, we will conduct a systematic narrative synthesis of gender differences, if reported, that summarizes key themes (e.g., related to intervention types and diagnostic subgroups), based on lists of common overlapping themes and sub-themes across the studies, created by two independent reviewers. Second, for the each primary and secondary outcome measure, we will conduct an IPDMA of treatment × gender interactions [49, 50], which is the recommended assessment of how treatment effects vary across patient subgroups. We chose a two-staged approach to allow for the potential inclusion of RCTs that do not agree to share their IPD data, but are willing to provide the required interaction estimates.

In the first stage of the IPDMA, we will fit linear regression models with restricted maximum likelihood (REML) estimation to assess the treatment (intervention vs. control) by gender (men vs. women) interactions, and their variances, of post-treatment scores within each trial, on a modified intention-to-treat basis. The models will include treatment, gender, and the interaction, and adjust for the baseline scores (corresponding to an analysis of covariance approach). The modified intention-to-treat (ITT) population for each included trial will consist of every randomized participant with known gender, treatment, and FED group for which at least a baseline score of FED-related pathology is available. Missing post-treatment and follow-up primary outcomes will be imputed by the last observation carried forward (LOCF) method. For missing secondary outcomes, LOCF may be applied if previous datapoints are available (e.g., BMI). Otherwise, the patient record will be excluded from the respective secondary outcome analysis.

In the second stage, estimates obtained for within-trial interactions for each trial will be combined using an inverse-variance weighted, random-effects meta-analysis model (allowing for between-study heterogeneity in the treatment x gender interaction) with REML estimation. To account for potential heterogeneity in baseline co-variates, we will repeat the previous analysis steps with the subgroup of studies that report these covariates, and compare the further adjusted results to the primary findings as a sensitivity analysis.

Additional exploratory trial-level moderations of treatment x gender interactions based on, for example, diagnostic group, setting-related factors (i.e., in- vs. outpatient treatment, group vs. individual therapy), and treatment approaches (e.g., CBT, psychodynamic therapies), will be examined using meta-regression. We will also estimate the effects separately for men and women, respectively. A two-tailed α < 0.05 will be applied to significance testing.

Results of the IPDMA will be presented as forest plots for the different interventions and FED diagnoses (AN, BN, BED, OSFED, others). Statistical heterogeneity will be examined for aggregate data using the *I*^*2*^ statistic, with *I*^*2*^ ≥ 50% deemed moderate, and *I*^*2*^ ≥ 75% deemed high heterogeneity.

To determine whether the two-staged IPDMA of treatment x gender interactions is sufficiently powered to detect gender differences (or interpret comparability), we conducted an a priori, simulation-based power analysis [51] using the Kovalchik and Cumberland method [52]. This method requires summary data of intervention outcomes, gender distribution for intervention and control groups, and group sizes, which were readily available for nine published RCTs which were pledged to the IPDMA during drafting the funding application. This simulation showed that this subset of studies [53–61] can already detect, for example, small- to medium-sized differences between gender groups in treatment-induced changes on a standard measure of FED psychopathology.

Throughout the review process, we will hold regular stakeholder meetings, comprised of a patient advisory board of 6 to 10 men who underwent FED treatment [62]. Stakeholders will provide input on findings and goals, and discuss and vote on the wording of a series of statements relating to clinical implications, which will be included in the published review.

### Meta-bias(es)

For the assessment of publication biases, contour-enhanced funnel plots with interaction effects on the horizontal axis and standard errors (SE) on the vertical axis, and tests of funnel plot asymmetry will be inspected. We will perform regression-based sensitivity analyses to assess whether the results are robust by excluding trials assessed as high risk of bias, and to assess the impact of missing data due to missing responses from data requests. As an additional sensitivity analysis for underlying assumptions, we will further compare the results of the two-staged IPDMA against a one-stage approach, using a generalized linear model with stratified, by-trial parameters for all effects outside the interaction term [50]. Reporting of the results will conform to the Preferred Reporting Items for Systematic Reviews and Meta-analyses of Individual Participant Data (PRISMA-IPD) Statement [63, 64].

### Confidence in cumulative evidence

The overall quality of evidence will be rated by two reviewers independently for the primary outcomes according to the Grading of Recommendations Assessment, Development, and Evaluation (GRADE) approach [65]. The quality will be graded on the following aspects: random sequence generation, allocation concealment, outcome completeness, and completeness of reporting. Quality assessment of both reviewers will be compared, and disagreements will be resolved by consulting the principal investigator. Results of the quality and risk-of-bias assessment will be included as moderator in the analyses.

## Discussion

Reports of suboptimal care and the underrepresentation of men in eating disorders research challenge whether current treatment recommendations apply towards men, creating uncertainty for clinicians and delaying the provision of care. Indeed, girls and women constitute a majority within RCT samples, severely limiting conclusions about FED treatment effects in men. As men’s under-representation in FED research may have inadvertently facilitated the development of treatments geared toward FED presentation in girls and women [35], systematic, gender-based comparisons of FED treatment effects are vital for ensuring optimal health care delivery, and identifying areas in need of improvement.

The IPDMA approach has been hailed as the “gold standard” of systematic review methodology, as it allows for more powerful and flexible analyses of both subgroups and outcomes. The planned IPDMA will thus establish evidence for either gender differences in treatment effects (prompting tailored recommendations and/or improving treatment approaches) or their equivalence (ensuring clinicians about the applicability of current treatment implementations), providing guidance on how to ensure optimal health care delivery, and for identifying areas in need of further research and improvement. If gender-specific differences are identified, we will develop clear recommendations on how to adapt existing treatment strategies.

## Supplementary Information

Additional file 1: Medline Search Example (via PubMeD).

## Data Availability

Meta-analysis coding sheets, coded data, codebooks, analysis syntax as well as any other non-confidential documents created as part of the systematic review will be made publicly available using the Open Science Framework (https://osf.io/) repository, adhering to FAIR principles. Data sharing is not applicable to this protocol as no datasets were generated or analyzed.

## Abbreviations

AN: Anorexia nervosa
ARFID: Avoidant/restrictive food intake disorder
BED: Binge-eating disorder
BN: Bulimia nervosa
CBT: Cognitive Behavioral Therapy
DALY: Disability-adjusted life year
DBT: Dialectical Behavioral Therapy
DSM: Diagnostic and Statistical Manual of Mental Disorders
EDRS: Eating Disorders Research Society
FAIR: Findability, Accessibility, Interoperability, and Reuse of digital assets
FBT: Family-based therapy
FED: Feeding and eating disorders
GRADE: Grading of Recommendations Assessment, Development, and Evaluation
ICD: International statistical Classification of Diseases and related health problems
IPDMA: Individual Patient Data Meta-Analysis
IPT: Interpersonal therapy
MeSH: Medical Subject Headings
OSFED: Other Specified Feeding or Eating Disorder
PRESS: Peer Review of Electronic Search Strategies
PT: Psychodynamic therapy
RCT: Randomized controlled trial
RoB: Risk-of-bias
