# Supplementary material for: Do we need to “man up” feeding and eating disorders treatments? Protocol for a systematic review and individual patient data meta-analysis of gender effects on intervention outcomes
Source: Syst Rev. 2025 Dec 24;15:31. doi: 10.1186/s13643-025-03041-5 (PMC12838484; doi:10.1186/s13643-025-03041-5)
Supplement: Supplementary file 1 — Additional file 1: Medline Search Example (via PubMeD). [file 13643_2025_3041_MOESM1_ESM.docx]

Medline Search Example (via PubMeD):

((Feeding and Eating Disorders [mh] OR Anorexia Nervosa [mh] OR

Avoidant Restrictive Food Intake Disorder [mh] OR Binge-Eating Disorder [mh] OR Bulimia Nervosa [mh] OR Diabulimia [mh] OR Feeding and Eating Disorders of Childhood [mh] OR Food Addiction [mh] OR Night Eating Syndrome [mh] OR Orthorexia Nervosa [mh] OR Pica [mh] OR Relative Energy Deficiency in Sport [mh] OR Female Athlete Triad Syndrome [mh] OR Rumination Syndrome [mh])

OR

((eating disorder) OR (anorex* ) OR (bulimi* ) OR (binge eating) OR (binge-eating) OR (EDNOS) OR (OSFED) OR (pica) OR (rumination disorder) OR (restrictive food intake disorder) OR (ARFID) OR (unspecified feeding or eating disorder) OR (UFED) OR (muscle dysmorphia) OR (orthorexi*)))

AND

((Psychotherapy [mh] OR Counseling [mh] OR Self-Help Groups [mh] OR Mental Health Services [mh] OR Psychosocial Intervention [mh] OR Internet-Based Intervention [mh])

OR

((intervention) OR (therap*) OR (program*) OR (treatment) OR (counselling) OR (counseling) OR (cognitive behavio*) OR (CBT) OR (behavio* therap*) OR (dissonance) OR (acceptance) OR (dialectical) OR (psychodynamic) OR (interpersonal) OR (mindfulness) OR (compassion) OR (family therap*) OR (family-based*) OR (self-help) OR (self help) OR (psychodynamic therap*)))

AND

((Treatment Outcome [mh] OR Patient Outcome Assessment [mh] OR Body Image [mh] OR Feeding Behavior [mh] OR Psychopathology [mh])

OR

((eating) OR (disordered eating) OR (overeating) OR (binging) OR (eating pathology) OR (eating symptom*) OR (emotional eating) OR (eating psycho*) OR (purg*) OR (dietary restraint) OR (restrict*) OR (diet*) OR (compulsive) OR (body image) OR (body dissatisfaction) OR (body satisfaction) OR (body esteem) OR (body appreciation) OR (appearance) OR (body functionality) OR (body preoccupation) OR (body shame) OR (body awareness) OR (body anxiety) OR (shape concern) OR (shape dissatisfaction) OR (weight concern) OR (weight dissatisfaction) OR (muscular*) OR (muscle) OR (body ideal*) OR (body surveillance) OR (drive thinness) OR (EDI) OR (EDE-Q)))

AND

((randomized controlled trial[pt]) OR (controlled clinical trial[pt]) OR (randomized[tiab] OR randomised[tiab]) OR (placebo[tiab]) OR (randomly[tiab]) OR (trial[tiab]) OR (groups[tiab])) NOT (animals[mh] NOT humans[mh])
